# Anti-TNF-α Compounds as a Treatment for Depression

**DOI:** 10.3390/molecules26082368

**Published:** 2021-04-19

**Authors:** Sarit Uzzan, Abed N. Azab

**Affiliations:** 1 Department of Clinical Biochemistry and Pharmacology, School for Community Health Professions—Faculty of Health Sciences, Ben-Gurion University of the Negev, P.O. Box 653, Beer-Sheva 8410501, Israel; sarituzzan@gmail.com; 2 Department of Nursing, School for Community Health Professions—Faculty of Health Sciences, Ben-Gurion University of the Negev, P.O. Box 653, Beer-Sheva 8410501, Israel

**Keywords:** bipolar disorder, depression, inflammation, pentoxifylline, TNF-α, TNFR

## Abstract

Millions of people around the world suffer from psychiatric illnesses, causing unbearable burden and immense distress to patients and their families. Accumulating evidence suggests that inflammation may contribute to the pathophysiology of psychiatric disorders such as major depression and bipolar disorder. Copious studies have consistently shown that patients with mood disorders have increased levels of plasma tumor necrosis factor (TNF)-α. Given these findings, selective anti-TNF-α compounds were tested as a potential therapeutic strategy for mood disorders. This mini-review summarizes the results of studies that examined the mood-modulating effects of anti-TNF-α drugs.

## 1. Mood Disorders

Millions of people around the world suffer from psychiatric illnesses, causing unbearable burden and immense distress to patients and their families [1]. Moreover, psychiatric disorders are associated with extensive financial costs to patients, the health care system and society in general [2,3]. Patients with mood disorders such as bipolar disorder and depressive disorders are of a higher likelihood to suffer from suicidal death and various comorbidities, leading to increased mortality rates in comparison to matched-healthy subjects [4,5,6]. The lifetime prevalence of bipolar disorder in the general population is between 0.7–1.5% [7,8] and that of depressive disorders is between 10–20% [1,9]. These estimations likely depict only a fraction of the true numbers, suggesting that there are presumably myriads of concealed and undiagnosed cases, and acknowledges that there is societal and cultural variance in recognition and interpretation of psychiatric symptoms [1,10].

Bipolar disorder is recognized as one of the most complex and difficult-to-treat psychiatric illnesses. Patients with bipolar disorder suffer alternating periods of mania and depression [11,12]. Mania is characterized by euphoric mood, impaired judgment, hyperactivity and excitement, increased erotic thoughts and engagement in sexual activity, among other features [11,12].

Depression is a rampant and devastating mental disorder [1,9], and is more prevalent in women than in men [1]. Melancholy is the primary feature/manifestation of depression [13,14,15,16]. Patients with depression may have alternative or accompanying symptoms including anxiety, low self-esteem, changes in appetite, social isolation, diminished interest in hedonic activities, insomnia or hypersomnia, and suicidal thoughts and/or attempts, among others [13,14,15,16]. Expectedly, the severity of symptoms and duration of depressive episodes vary significantly and, understandably, depressive episodes can impact even the most basic aspects of patients’ lives. Occasionally, depression presents without a known triggering cause. However, sometimes a prominent emotional stimulus, such as a death of a close relative, precedes the inception of depression.

The most widely used treatment strategy for bipolar disorder is pharmacotherapy [11,12,17]. Other approaches include electroconvulsive therapy [18,19] and cognitive behavioral therapy [20]. Similarly, pharmacotherapy, psychotherapy and electroconvulsive therapy are the three most frequently used treatments for depressive disorders [17,18,21,22,23,24]. Among these, pharmacotherapy is the most common and it includes a wide variety of medications [23,24]. The treatment of depressive disorders is dictated by a number of factors including: (i) risk of suicide, (ii) the patient’s ability to understand and follow instructions (adherence to treatment), (iii) level of supportive resources, (iv) level of encountered stressors, and, (v) level of functional impairment [17,24].

The availability of abundant and diverse medication options available for the treatment of mood disorders notwithstanding, a high proportion of patients present a poor response to treatment [11,12,14,17,22,23,24]. Moreover, many patients suffer a plethora of unpleasant side effects (some of which may be severe and irreversible) further encouraging poor compliance to treatment [11,12,14,17,22,23,24,25,26,27]. These limitations accentuate the necessity for new treatment strategies for mood disorders in an effort to supply hope for additional sub-groups of patients.

## 2. Tumor Necrosis Factor (TNF)-α

**Figure 1:**
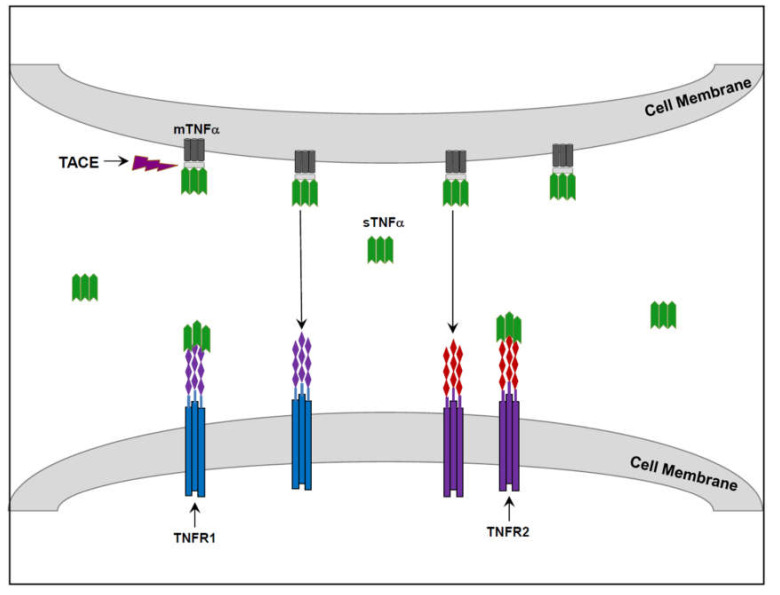
TNF-α and TNF-α Receptors. Transmembrane TNF-α (mTNF-α) undergoes proteolytic cleavage by TNF-α-converting enzyme (TACE) which generates the soluble form of the protein (sTNF-α). Both mTNF-α and sTNF-α are biologically active; they bind to and activate TNF receptor (TNFR) 1 and TNFR2. Arrows indicate that mTNF-α is also capable of activating TNFR1 and TNFR2.

TNF-α is a multi-functional cytokine which plays central roles in numerous physiological as well as pathological processes in mammals [28,29,30,31]. It was recognized early on for its ability to induce necrosis of tumor cells [32], but was subsequently associated with plentiful biological functions [28,29,30,31]. TNF-α is synthetized and secreted mainly by macrophages though several cell types (including glia cells and neurons in the brain) are capable of producing it [28,29,30,31,32,33,34,35]. Newly synthesized TNF-α localizes in cell membrane until it undergoes proteolytic cleavage by TNF-α-converting enzyme, which releases the soluble form of the protein [36,37] (see Figure 1 for illustration). Both the transmembrane and the soluble form of the protein are biologically active—binding to and activating TNF receptor 1 (TNFR1) as well as TNFR2 [30,31,38,39] (Figure 1). TNFR1 and TNFR2 share some similar functions (e.g., advancement of immune defense mechanisms, induction of inflammation, and promotion of cell proliferation and survival) but, they also have distinct, sometimes opposite, biological activities [30,31,38,39]. Principally, TNFR1 is connected to pathological processes such as inflammation, apoptosis and necrosis, while TNFR2 is mostly linked to physiological responses such as host defense, tissue repair and regeneration [30,31,38,39]. However, delineating these receptors with distinctive pathological versus physiological tasks would be an over-simplification of a more complex biological reality.

Thorough research has indicated TNF-α to be mostly linked to immune and inflammatory functions [30,31]. It has also been associated with cancer pathophysiology [29]. It is involved in various immune and inflammatory responses (usually acting as a pro-inflammatory mediator) contributing to host defense [30,31,38,39]. Under certain conditions, TNF-α facilitates apoptosis and cell death especially in cancer cells [29,30,31,38,39]. Nevertheless, and despite its common association with pathological conditions, TNF-α plays a crucial role in numerous physiological processes, particularly in the central nervous system (CNS—the brain and the spinal cord) [28,39]. For example, in the brain, TNF-α has a direct impact on neuronal function and survival, regulating production and secretion of neurotransmitters, controlling synaptic transmission, and contributing to myelin synthesis and preservation [28,39,40,41,42,43,44,45]. TNF-α was found to increase the permeability of the blood-brain barrier (BBB) which is accompanied by depressive behavior [46,47,48]. Dysfunction of the BBB hastens the penetration of inflammatory mediators and peripheral immune cells into the CNS leading to behavioral abnormalities and mood disorders [49,50]. Thus, taking into account the various crucial functions of TNF-α, it is expected that disruption of its activity would cause profound biological consequences, including alteration of neurological function. 

## 3. Brain Inflammation, TNF-α and Mood Disorders

The CNS consists of two main types of cells: neurons and glia cells [33,34]. There are three types of glia cells: astrocytes, microglia, and oligodendrocytes [33,34]. The role of microglia cells in the CNS is comparable to that of macrophages in peripheral tissues. Astrocytes have important immune-inflammatory roles, and support the function and survival of neurons [33,34,51]. Oligodendrocytes produce myelin, the insulating substance that surrounds nerve cell axons. Microglia and astrocytes are involved in various neuro-inflammatory processes and are associated with numerous CNS pathologies [28,34,35,51,52,53,54]. Despite the presence of the BBB, the activity of the “peripheral” immune system still manages to impact the CNS. It has been consistently recognized that illnesses associated with systemic inflammation (e.g., rheumatoid arthritis and coronary artery disease) frequently present with behavioral abnormalities and symptoms of depression. Systemic inflammatory responses to infectious agents affect brain function and, in turn, evoke significant changes in behavior [54]. This association has revealed itself to be more than just a speculation, as even early studies suggested that dysregulation of the immune system may lead to depression [55,56]. Subsequently, many studies reported that immune-dysregulation and inflammation contribute to the pathophysiology of mood disorders. It was found that patients with depression had elevated levels of pro-inflammatory markers [57,58,59,60,61,62,63,64,65,66,67,68,69,70], while levels of anti-inflammatory mediators were either comparable [71,72] or lower [73] than those in control subjects. Bipolar patients were also reported to have abnormal levels of various inflammatory mediators [59,72,74,75,76,77,78,79,80,81,82,83,84,85]. In particular, numerous studies reported that TNF-α levels are elevated in patients with major depression [56,59,60,61,62,69,70,86] and bipolar disorder [59,72,74,75,76,78,79,80,81,82,83,84,85]. Abnormalities in TNF-α levels have been shown to influence the severity of psychiatric symptoms and response to treatment. For example, a recent study showed that elevated baseline plasma TNF-α levels in patients with major depression may predict a better improvement in intensity of suicidal thoughts [86]. Patients with bipolar disorder [87] and depression [88] were reported to have altered levels of TNFR1 and TNFR2, respectively. Interestingly, the latter two studies [87,88] did not demonstrate abnormal TNF-α levels among their population. However, despite the large body of data attesting for alterations in inflammatory mediator levels among patients with mood disorders, some studies reported opposite findings [80,85].

Furthermore, the “inflammation hypothesis” of mood disorders was strengthened by data that showed that psychotropic drugs possess anti-inflammatory effects. Antidepressants, mood stabilizers and antipsychotic drugs were reported to have anti-inflammatory effects which may contribute to their therapeutic efficacy [67,70,89,90,91,92,93,94,95,96,97,98,99,100,101,102,103,104,105,106,107]. For example, Li et al. [98] reported that the mood stabilizer lithium reduced levels of TNF-α in patients with acute manic episodes. Valproate, another mood stabilizer, reduced the secretion of interleukin (IL)-6 and TNF-α production in vitro [108]. Similarly, various antidepressants were found to have potent anti-inflammatory effects [68,70,99,109,110]. This outcome is exemplified by the selective serotonin reuptake inhibitor fluoxetine which significantly decreased plasma IL-6 levels in patients with acute depression [111]. Antipsychotic drugs also exhibited anti-inflammatory effects [89,93,94,102,104,106]. This response can be seen in second generation antipsychotic drugs that decreased lipopolysaccharide-induced synthesis of IL-6 and TNF-α and increased the levels of the anti-inflammatory cytokine IL-10 in mice [102]. In contrast to these findings, some studies showed that psychotropic drugs exhibit pro-inflammatory effects in certain circumstances [64,89,104,107,112,113,114,115,116].

Additional support for the inflammation hypothesis of mood disorders came from studies that showed that treatment with various anti-inflammatory/immune-modulating drugs reduced symptom severity and improved conditions of patients with mood disorders [58,117,118,119,120,121,122,123]. Mainly, selective cyclooxygenase-2 inhibitors (e.g., celecoxib) were found beneficial as add-on therapy to psychotropic drugs in patients with mood disorders [58,120]. Nevertheless, here too, studies published negative findings regarding the effectiveness of anti-inflammatory/immune-modulating medications as a treatment for mood disorders [124,125]. Among the various anti-inflammatory drugs that have been explored as a potential treatment for mood disorders, selective TNF-α antagonists were given special attention. The following section summarizes the mood-modulating effects of clinically used anti-TNF-α compounds.

## 4. Anti-TNF-α as a Treatment for Mood Disorders

As summarized above, a large body of data suggested that out of the inflammatory mediators that have been linked to the pathophysiology of mood disorders, TNF-α in particular exhibited a seemingly significant role [56,57,58,59,69,70,71,78,80,84,86,90,126]. This was the basis for investigating the mood-modulating effects of selective anti-TNF-α compounds. Several selective anti-TNF-α compounds were developed and introduced for clinical use, typically for the treatment of immune-inflammation-related disorders such as rheumatoid arthritis, ankylosing spondylitis, psoriasis, inflammatory bowel diseases (e.g., Crohn’s disease), and hidradenitis suppurativa, among others [30,127,128,129,130,131,132,133,134,135,136,137,138,139,140]. The following paragraphs summarize the results of studies that tested the mood-modulating effects of anti-TNF-α compounds.

### 4.1. Search Strategy

The search strategy was based on surveying the following electronic databases for inclusive criteria: PubMed, Web of Science, and Google Scholar, for English language papers published in peer-reviewed journals reporting on the use of anti-TNF-a compounds in subjects with mood disorders. The customized search was restricted to the years 1990 (the year when the first report on the anti-TNF-a activity and beneficial therapeutic effects of infliximab was published [141]) to 2020. The search field contained the name of each compound, including: infliximab, etanercept, onercept, adalimumab, golimumab, humicade, certolizumab pegol, and pentoxifylline; together with each of the following keywords: depression, melancholia, depressive disorder, mania, bipolar disorder, manic-depressive illness. The search strategy resulted in many hits that were irrelevant to the purpose of the article. On the other hand, no relevant papers reporting on the effects of onercept, golimumab, humicade and certolizumab pegol in subjects with mood disorders were found. We included most relevant papers reporting on animal studies and almost all papers reporting on studies conducted in human subjects, because the latter were the main focus of the manuscript.

### 4.2. Infliximab

**Figure 2:**
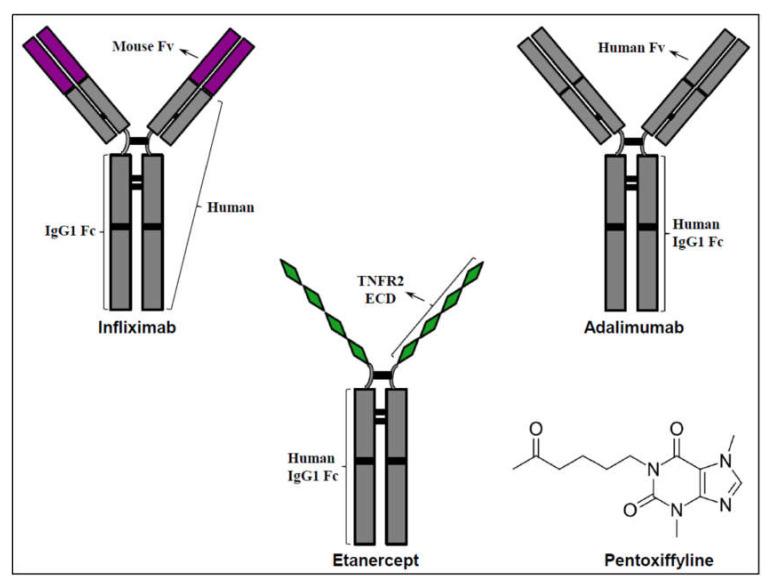
TNF-α Antagonists. Clinically used selective TNF-α antagonists include recombinant TNF-α-specific monoclonal antibodies such as infliximab and adalimumab, and recombinant fusion proteins of TNFR such as etanercept which is a TNFR2 fusion protein. Pentoxifylline is a methylxanthine drug which exerts several pharmacological effects including potent inhibition of TNF-α activity (i.e., it is not a selective TNF-α antagonist). Abbreviations: ECD—extracellular domain, Fc—fragment crystallizable region, Fv—variable fragment, IgG—immunoglobulin G, TNFR2 – TNF-α receptor 2.

Infliximab is a chimeric TNF-α-specific neutralizing monoclonal antibody consisting of a human IgG Fc region and a murine Fv region (see Figure 2 for illustration). It is recognized as a potent selective TNF-α antagonist with powerful neutralizing effects against soluble TNF-α and, to a lesser extent, on transmembrane TNF-α [133,142,143,144]. Infliximab is capable of binding to both monomeric and trimeric forms of soluble TNF-α. Each infliximab molecule can bind to two TNF-α molecules, while a single TNF-α homotrimer can bind to up to three infliximab molecules [133,142,143,144]. Infliximab is administered intravenously and thus has a maximized (100%) bioavailability; it has a low clearance rate (~ 11 mL/hour) and a plasma half-life of nearly 8–10 days [133,143]. Infliximab has been used for the treatment of various rheumatoid and inflammatory-associated diseases such as rheumatoid arthritis, psoriasis, ankylosing spondylitis, and Crohn’s disease, among others [30,133]. Several studies examined the effects of infliximab on depressive symptoms among patients with Crohn’s disease [134,135] and ankylosing spondylitis [136,145,146] revealing encouraging results. Animal studies also demonstrated an antidepressant-like effect for infliximab [147,148]. Raison et al. [149] evaluated the antidepressant effect of infliximab in patients with treatment-resistant depression. Sixty patients were randomly allocated to receive either infliximab (*n* = 30) or a placebo (*n* = 30). Infliximab showed a significant therapeutic effect—mitigated depressive symptoms—but only in patients who had increased levels of inflammatory markers [149]. Consistent with these results, a recent meta-analysis study which evaluated the antidepressant efficacy of infliximab revealed that it was effective exclusively in patients with elevated levels of inflammatory markers such TNF-α and C-reactive protein [150]. The efficacy of infliximab was also tested in patients with bipolar depression [151,152,153,154]. McIntyre et al. [151] conducted a randomized, double-blind, placebo-controlled trial in which 29 patients were treated with infliximab and 31 patients with a placebo. Twelve weeks of infliximab treatment did not cause a significant reduction in severity of depressive symptoms. Only in a sub-group of patients with a history of childhood physical abuse infliximab (as compared to the placebo) led to a significant depletion in depressive symptoms [151]. Lee et al. [152] conducted a randomized, double-blind trial of adjunctive treatment with infliximab (together with standard pharmacotherapy) and a placebo for 12 weeks in patients with bipolar depression. They reported a significant improvement in a measure of anhedonia in infliximab-treated patients; however, the positive effect was short-lived and did not show sustainable positive results, dissipating within six weeks after the final infusion of the drug. Mansur et al. also reported positive therapeutic effects of infliximab on depressive symptoms [153] and cognitive function [154] in patients with bipolar depression. A recent study by the same group of investigators also demonstrated beneficial effects of infliximab on bipolar patients [155]. In a 12-week, randomized, double-blind trial, infliximab treatment was associated with a significant decrease in prefrontal levels of glutamate and a cognitive improvement in patients with bipolar depression [155]. Together, these findings (see summary of the findings in Table 1) suggest that infliximab produces antidepressant effects in particular sub-groups of depressive patients.

### 4.3. Etanercept

Etanercept is a human recombinant fusion protein of TNFR2 that neutralizes/inhibits TNF-α activity [30] (Figure 2). It is regarded as a less powerful TNF-α antagonist when compared to infliximab, but similarly to infliximab, it has a much stronger antagonizing effect against soluble TNF-α than transmembrane TNF-α [133,142,143,144]. Etanercept binds only to the trimeric form of soluble TNF-α and each etanercept molecule is capable of binding to one TNF-α molecule [133,142,143,144]. Etanercept is administered subcutaneously and has a bioavailability of nearly 75%; it has a relatively high but varying clearance rate (80–240 mL/hour) and a plasma half-life of 3–5.5 days [133,143]. Early pre-clinical studies showed that etanercept reduced depressive-like behavior in rats [156,157]. More recently, a study in rats showed that etanercept significantly decreased depressive-like behavior and improved cognitive function [158]. Similarly, a study in mice showed that etanercept exerted a potent antidepressant-like effect and an anxiolytic-like effect [159]. In line with these pre-clinical results, etanercept was found to significantly decrease the severity of fatigue, depression and anxiety symptoms among patients with psoriasis (Table 1) [137,138,160,161]. Moreover, non-randomized trials showed that addition of etanercept to standard therapy significantly reduced depressive and anxiety symptoms among patients with psoriasis [162,163,164] and rheumatoid arthritis [165,166]. For example, a prospective cohort study by Yang et al. [167] demonstrated that addition of etanercept to standard treatment was associated with a sustained significant reduction in depression and anxiety symptoms in psoriasis patients. In contrast to these findings, a study in patients with rheumatoid arthritis found that addition of etanercept to methotrexate (an immune-modulating drug) did not significantly improve depressive and anxiety symptoms [139]. Collectively, these results suggest that etanercept exhibits antidepressant and anxiolytic effects at least in some sub-groups of patients.

### 4.4. Adalimumab

Adalimumab is another human TNF-α-specific neutralizing monoclonal antibody (Figure 2). It has similar pharmacokinetic properties to infliximab. Each adalimumab molecule can bind to two TNF-α molecules, while a single TNF-α homotrimer can bind to up to three adalimumab molecules [133,142,143,144]. Adalimumab is administered subcutaneously and has a bioavailability of nearly 65%; it has a low clearance rate (~12 mL/hour) and a long but variable plasma half-life ranging from 10 to 20 days [133,143]. Randomized and non-randomized clinical trials showed that adalimumab exerts antidepressant and anxiolytic effects when administered to patients with chronic physical illnesses such as Crohn’s disease [140], psoriasis [128,129,168,169,170] and hidradenitis suppurativa [130] (Table 1). To the best of our knowledge, the mood-modulating effects of adalimumab have not been directly tested in psychiatric patients with mood disorders.

### 4.5. Pentoxifylline

Pentoxifylline is a methylxanthine drug (Figure 2) that for many years has been used for the treatment of different clinical conditions such as peripheral vascular disease [171,172], idiopathic and ischemic cardiomyopathy [173,174,175], coronary artery disease [176], chronic kidney disease [177], alcoholic hepatitis [178], among other illnesses [171,179,180]. Pentoxifylline is administered orally and has a relatively high bioavailability, depending on the used formulation [160]. It has a low binding rate to plasma proteins (minimizing the chance for drug-drug interactions) and distributes vastly throughout body tissues, extending to the brain. Pentoxifylline undergoes extensive metabolism (mainly through reduction and oxidation) and has a short plasma half-life ranging between 1 to 4 h, again, depending on the used formulation [160]. The therapeutic efficacy of pentoxifylline in the treatment of peripheral vascular disease seems to be derived from its ability to improve the deformability of red blood cells, decrease blood fibrinogen levels and inhibit platelet aggregation [172]. Moreover, pentoxifylline inhibits the enzyme phosphodiesterase [181]. In the context of the present article, pentoxifylline is recognized as a potent inhibitor of TNF-α [173,174,175,176,177,179,181,182,183,184,185,186]. Numerous studies showed that pentoxifylline inhibits the production of TNF-α in vitro and in vivo (in animals and humans) [173,174,175,176,177,179,181,182,183,184,185,186]. Thus, pentoxifylline is regarded as a strong non-selective TNF-α inhibitor (as it exerts other pharmacological properties).

Owing to the large body of data which linked TNF-α to the pathophysiology of depression, many pre-clinical studies have investigated the antidepressant potential of pentoxifylline [182,183,187]. Bah et al. [187] demonstrated that pentoxifylline exerted antidepressant-like effects in rats that were subjected to an experimental model of myocardial infarction. Pentoxifylline significantly increased sucrose preference and significantly decreased immobility time (both indicative of an antidepressant-like effect) in the forced swim test in post-infarction rats [187]. Mohamed et al. [182] observed that treatment with pentoxifylline for three weeks significantly increased sucrose preference in rats that were subjected to a chronic mild stress protocol. The chronic mild stress paradigm is used to induce depressive-like phenotypes in animals. Another study showed that pentoxifylline significantly decreased immobility time in rats that were exposed both to an inflammatory stimulus (lipopolysaccharide) and chronic mild stress [183]. Collectively, these studies [182,183,187] (among others) demonstrated that pentoxifylline has strong antidepressant-like effects in various behavioral models including the sucrose preference test and the forced swim test [182,183,187]. Consistent with these positive pre-clinical results, a randomized, double-blind, placebo-controlled clinical trial showed that adjunctive pentoxifylline treatment was associated with a significant anti-depressive effect [188]. Addition of pentoxifylline (400 mg/day) to escitalopram (20 mg/day) for 12 weeks significantly reduced depressive symptoms in patients with major depression [188]. Moreover, pentoxifylline caused a significant decrease in plasma TNF-α and IL-6 levels (suggestive of a potent anti-inflammatory effect) and a significant increase in plasma serotonin and brain-derived neurotrophic factor levels (suggestive of favorable behavioral/neuroprotective biochemical effects) [188]. These encouraging findings underscore the need for more randomized trials of pentoxifylline in patients with mood disorders.

## 5. Summary

Several clinical trials attested for the antidepressant efficacy of anti-TNF-α compounds (in patients with medical illnesses, major depression, or bipolar depression) [70]. Selective TNF-α antagonists such as infliximab and etanercept showed favorable neurological/antidepressant effects in specific sub-groups of patients. However, it is important to emphasize that most of the available data regarding the antidepressant effects of selective TNF-α antagonists is derived from studies in non-psychiatric patients (i.e., patients with inflammatory-associated diseases who presented depressive symptoms). Moreover, some evidence suggests that there is no connection between anti-TNF-α therapy and improvement in mood symptoms [139,150,151]. Therefore, new randomized, placebo-controlled clinical trials are necessary for direct examination of the mood-modulating effects of TNF-α antagonists in patients with mood disorders. In this regard, recently, concerns have been raised regarding the efficacy of selective TNF-α antagonists as a therapeutic strategy for mood disorders [139,151,189,190]. It is important to mention that most clinically available anti-TNF-α compounds possess low-to-null ability to cross the BBB, mainly due to their large molecular weight [191,192,193]. This suggests that the reported beneficial behavioral (antidepressant) effects of these compounds are derived from peripheral inhibition of TNF-α activity rather than a direct effect on the brain. Potent peripheral inhibition of TNF-α activity may be sufficient for diminishing brain inflammation. Therefore, it is important to continue studying the therapeutic mechanism of action and effectiveness of selective TNF-α antagonists as a treatment for mood disorders.

## Figures and Tables

**Table 1:** Summary of clinical trials reporting on the mood-modulating effects of anti-TNF-α compounds in patients with various disease conditions.

Compound	Study Design	Sample Size (Total)	Disease Condition	Type of Comparison (Follow-Up Duration) *	Effect of Treatment	Ref.
Infliximab	Prospective, non-randomized trial	*n* = 100	Crohn’s disease	All patients were treated with infliximab + standard therapy (4 weeks)	Significant decrease in the proportion of depressed patients	[134]
	Prospective, non-randomized trial	*n* = 14		All patients were treated with infliximab + standard therapy (4 weeks)	Significant reduction in depressive symptoms	[135]
	Prospective, non-randomized trial	*n* = 29	Ankylosing spondylitis	All patients were treated with three doses of infliximab + standard therapy (6 weeks)	Significant reduction in depressive symptoms	[136]
	Randomized, placebo-controlled trial	*n* = 23		Standard therapy + placebo vs. standard therapy + infliximab, followed by infliximab-only treatment (54 weeks)	Significant reduction in depressive symptoms	[146]
	Randomized, double-blind, placebo-controlled trial	*n* = 60	Major depressive disorder (treatment-resistant)	Antidepressant(s) or medication free + placebo vs. antidepressant(s) or medication free + infliximab (12 weeks)	Overall, no significant difference between groups. Infliximab significantly decreased depressive symptoms in a sub-group of patients with high baseline CRP levels	[149]
	Systematic review and meta-analysis of four randomized controlled trials	*n* = 152		Standard therapy + placebo vs. standard therapy + infliximab	Adjunctive infliximab treatment did not have a significant effect on depressive symptoms	[150]
	Randomized, double-blind, placebo-controlled trial	*n* = 60	Bipolar depression with higher inflammatory activity	Standard therapy + placebo vs. standard therapy + infliximab (12 weeks)	No significant difference between groups. Infliximab significantly decreased depressive symptoms in a sub-group of patients with a history of childhood physical abuse	[151]
	Randomized, double-blind, placebo-controlled trial	*n* = 60		Standard therapy + placebo vs. standard therapy + infliximab (12 weeks)	Adjunctive infliximab treatment led to a significant although transient anti-anhedonic effect	[152]
	Randomized, double-blind, placebo-controlled trial	*n* = 55	Bipolar depression	Standard therapy + placebo vs. standard therapy + infliximab (12 weeks)	Significant reduction in depressive symptoms	[153]
	Randomized, double-blind, placebo-controlled trial	*n* = 60		Standard therapy + placebo vs. standard therapy + infliximab (12 weeks)	Significant improvement in cognitive function (verbal memory)	[154]
	Randomized, double-blind, placebo-controlled trial	*n* = 33		Standard therapy + placebo vs. standard therapy + infliximab (12 weeks)	Significant improvement in cognitive function but no significant effect on depressive symptoms	[155]
Etanercept	Randomized, double-blind, placebo-controlled trial (phase 3)	*n* = 618	Psoriasis	Standard therapy + placebo vs. standard therapy + etanercept (12 weeks)	Significant decrease in depressive symptoms	[137]
	Prospective open-labeled trial (open-phase continuum of the study reported in reference # 137)	*n* = 591		Standard therapy + etanercept (84 weeks)	A sustained significant decrease in depressive symptoms	[138]
	Randomized, double-blind, placebo-controlled trial	*n* = 121		Standard therapy + placebo vs. standard therapy + etanercept (24 weeks)	Significant decrease in depressive symptoms	[160]
	Prospective, non-randomized trial	*n* = 85		Standard therapy + etanercept (24 weeks)	Significant reduction in depression and anxiety symptoms	[161]
	Prospective, non-randomized (open-labeled) trial	*n* = 2546		Standard therapy + etanercept given in two regimens—continues vs. interrupted (24 weeks)	Etanercept treatment (both regiments) led to a significant decrease in depressive symptoms	[162]
	Prospective, non-randomized (open-labeled) trial	*n* = 711		Standard therapy + etanercept given in two regimens—continues vs. interrupted (54 weeks)	Etanercept treatment (both regiments) led to a significant improvement in depressive symptoms	[163]
	Part 1: A randomized, double-blind, dose-adjusted trial; Part 2: Open-labeled trial	*n* = 752		Standard therapy + etanercept given in various regimens (24 weeks)	Significant reduction in depression and anxiety symptoms	[164]
Adalimumab	Randomized, double-blind, placebo-controlled trial (phase 3)	*n* = 499	Crohn’s disease	Standard therapy + adalimumab given in various regimens (56 weeks)	Significant decrease in depressive symptoms	[140]
	Randomized, double-blind, placebo-controlled trial	*n* = 96	Psoriasis	Standard therapy + placebo vs. standard therapy + adalimumab (12 weeks)	Significant decrease in depressive symptoms	[128]
	Prospective, non-randomized trial	*n* = 143		Standard therapy + adalimumab (24 weeks)	Significant reduction in depression and anxiety symptoms	[129]
	Randomized, double-blind, placebo-controlled trial	*n* = 828		Standard therapy + placebo vs. standard therapy + adalimumab (16 weeks)	Significant decrease in depressive symptoms	[168]
	Prospective, non-randomized trial	*n* = 32		Standard therapy + adalimumab (24 weeks)	Significant decrease in depressive symptoms	[169]
	Randomized, double-blind, placebo-controlled trial (phase 3)	*n* = 992		Standard therapy + placebo vs. standard therapy + adalimumab (and vs. standard therapy + guselkumab) (24 weeks)	Adalimumab significantly decreased depression and anxiety symptoms	[170]
	Randomized, double-blind, placebo-controlled trial (phase 2)	*n* = 154	Hidradenitis suppurativa	Standard therapy + placebo vs. standard therapy + adalimumab (16 weeks)	Adalimumab significantly decreased depressive symptoms in patients with high baseline pain score	[130]
Pentoxifylline	Randomized, double-blind, placebo-controlled trial	*n* = 100	Major depressive disorder	Escitalopram + placebo vs. escitalopram + pentoxifylline (12 weeks)	Significant decrease in depressive symptoms	[188]

* Type of comparison and follow-up duration are indicated in the table only if they were clearly mentioned in the reporting article. CRP denotes C-reactive protein.

## Data Availability

Not applicable.
